# Virtual brain and electroencephalography explain the variance of memory alterations in mild cognitive impairment

**DOI:** 10.1186/s13195-026-02114-4

**Published:** 2026-06-20

**Authors:** Anita Monteverdi, Matteo Cotta Ramusino, Francesca Conca, Alberto Augello, Chiara Totaro, Paolo A. Grasso, Anna Castelnovo, Roberta M. Lorenzi, Michele Terzaghi, Lisa M. Farina, Alfredo Costa, Anna Pichiecchio, Stefano F. Cappa, Claudia Gandini Wheeler-Kingshott, Fulvia Palesi, Egidio D’Angelo

**Affiliations:** 1 Digital Neuroscience Centre, IRCCS Mondino Foundation, Pavia, Italy; 2 Unit of Behavioral Neurology, IRCCS Mondino Foundation, Pavia, Italy; 3 University Institute of Advanced Studies (IUSS), Pavia, Italy; 4 Department of Brain and Behavioral Sciences, University of Pavia, Pavia, Italy; 5 Department of Child Neurology and Psychiatry, IRCCS Mondino Foundation, Pavia, Italy; 6 Department of Physics and Astronomy, University of Florence, Florence, Italy; 7 Sleep Medicine Unit, Neurocenter of Italian Switzerland, Ente Ospedaliero Cantonale (EOC), Lugano, Switzerland; 8 Faculty of Biomedical Sciences, Università Della Svizzera Italiana, Lugano, Switzerland; 9 Sleep Medicine Centre, IRCCS Mondino Foundation, Via Mondino, Pavia, Italy; 10 Advanced Imaging and Artificial Intelligence Center, IRCCS Mondino Foundation, Pavia, Italy; 11 IRCCS Istituto Auxologico Italiano, Milan, Italy; 12 NMR Research Unit, Department of Neuroinflammation, Queen Square Multiple Sclerosis Centre, UCL Queen Square Institute of Neurology, London, UK

**Keywords:** Excitatory/inhibitory balance, Mild cognitive impairment, Electroencephalography, Resting-state networks, Virtual brain modelling

## Abstract

**Background:**

Mild Cognitive Impairment (MCI) is a heterogeneous clinical condition characterized by a wide spectrum of cognitive and behavioural manifestations. Despite numerous studies, the link between neuropsychological performance and pathophysiological signatures of the disease—including Aβ and tau accumulation along with altered excitation/inhibition (E/I) balance and brain rhythms—remains elusive.

**Methods:**

Here Aβ/tau biomarkers were used to distinguish positive (MCI^+^- prodromal Alzheimer’s disease) and negative (MCI^−^) subjects in a cohort of 30 MCI patients (18 MCI^+^ and 12 MCI^−^). Virtual brain models based on high-field magnetic resonance imaging data were then developed to determine the inter-node coupling and E/I profile in resting-state networks, while node spectral information was obtained from source analysis of high-density electroencephalography (HD-EEG). Finally, virtual brains and HD-EEG parameters, creating brain digital twins of individual subjects, were correlated with cognitive performance.

**Results:**

While virtual brain simulations did not reveal E/I differences between MCI^+^ and MCI^−^, a positive correlation emerged between synaptic parameters of the limbic network and verbal episodic memory for both groups. EEG power spectral density revealed a lower high-frequency/low-frequency ratio in MCI^+^ largely due to a reduced alpha band in the default mode, limbic, attention, frontoparietal, visual and somatomotor networks. A strong correlation emerged between multimodal parameters and memory functions, supporting that brain digital twin simulations can effectively explain the variability of neuropsychological performance in MCI patients beyond the sensitivity of individual techniques alone. In particular, the combination of HD-EEG and virtual brain parameters explained more than 90% of variance for episodic memory patients’ scores, confirming the compound origin of memory performance involving network specific E/I levels and electroencephalographic activity acting in concert.

**Conclusions:**

This multimodal and multiparametric analysis combining virtual brain modelling with HD-EEG and molecular data enhances the stratification of MCI patients and could be used to develop digital biomarkers of progression to dementia, opening new perspectives for personalized prognosis and treatment.

**Supplementary Information:**

The online version contains supplementary material available at https://doi.org/10.1186/s13195-026-02114-4.

## Introduction

Mild Cognitive Impairment (MCI) is a heterogeneous condition characterized by a broad spectrum of cognitive alterations mostly affecting memory functions. MCI patients are at an increased risk of developing dementia, most often due to Alzheimer’s disease (AD) (annual conversion rate 6% in population settings to 11% in clinical settings) [1]. Identifying the neurophysiological alterations subtending the neuropsychological profiles of MCI patients could provide critical cues for a personalized diagnosis and the prediction of disease progression. Although neurophysiological studies reported correlations between Aβ and tau deposition and alterations of brain rhythms [2] and excitation/inhibition (E/I) balance [3–10], mechanistic models determining a link between these parameters, brain organization, and large-scale dynamics in individual patients are still missing.

The E/I balance, i.e., the dynamic interplay of glutamatergic excitation and GABAergic inhibition, is a core organizing principle of brain circuit function and plasticity [11–13]. When the mean excitatory and inhibitory inputs to neurons are balanced, i.e., they roughly cancel out, the neuronal firing is mostly driven by input fluctuations [11]. Therefore, E/I balance alterations impact large-scale brain dynamics and can be inferred by functional measurements like functional magnetic resonance imaging (fMRI), magnetoencephalography (MEG), and electroencephalography (EEG), using model inversion strategies [5, 6, 14–17]. Here, we used the virtual brain (TVB) [18, 19] modelling framework to assess brain network functional dynamic alterations in MCI patients. TVB combines structural and functional magnetic resonance imaging (MRI) data with mesoscopic models of neural activity (e.g., the Wong-Wang [10] neural mass model) associated to grey matter nodes (areas segmented on brain atlases); the simulated neuronal signal propagates between nodes through connected edges (fibres bundles obtained from MRI tractography) generating virtual brain models that simulate large-scale brain dynamics. TVB model inversion is performed through an optimization process against functional data (resting-state fMRI) yielding parameters of interest, including the coupling between nodes and the strength of NMDA and GABA receptor-mediated transmission and of recurrent excitation inside nodes. This information allows to estimate the E/I balance non-invasively in individual subjects [20, 21]. The role of E/I balance in the cascade of pathophysiological events along the AD continuum is increasingly recognized in literature. E/I disruption is not only the consequence of amyloid-beta deposition but also a driver of amyloid pathology [22]. The accumulation of Aβ and tau and E/I imbalance impact on neuronal activity manifesting, at the macroscopic level, as brain rhythms changes in patients [4] and even in cognitively healthy individuals positive to cerebrospinal fluid biomarkers [23].

Brain rhythms recorded with the electroencephalogram (EEG) reflect the bidirectional neuronal communication between cortical and subcortical structures [24]. The progression from MCI to AD dementia entails alterations of brain rhythms, with the EEG power decreasing in the alpha band and increasing in the delta-theta band (2–8 Hz) [22–25]. The link between molecular biomarkers (Aβ and tau) and brain rhythms has been demonstrated through studies that combine positron emission tomography (PET) imaging with EEG analysis [3, 4]. However, bridging the gap between neurophysiological mechanisms (e.g. biomarkers accumulation, E/I imbalance, brain rhythmic activity) and neuropsychological performance is still needed, especially in heterogeneous clinical conditions such as MCI. Here we performed high-density EEG (HD-EEG) recordings on the MCI patients and used standardized low-resolution brain electromagnetic tomography (sLORETA) [26] to obtain information about power spectral density (PSD) in the same brain regions considered as TVB nodes.

The E/I and PSD data could finally be used for a multimodal multiparametric characterization of circuit alterations in the MCI brain and to predict the neuropsychological performance of patients over multiple cognitive domains. We hypothesized that combining TVB-derived E/I parameters with HD-EEG spectral measures would better explain neuropsychological heterogeneity in MCI than either modality alone. A strong parameter correlation emerged with memory and verbal functions, supporting that brain digital twin simulations can effectively explain the variability of neuropsychological performance in MCI patient, and opening perspectives for a personalized classification and prediction of the progression to AD dementia.

## Methods

### Subjects

A total of 30 MCI patients (17 females, aged 73 ± 5 years) were recruited at the IRCCS Mondino Foundation (Pavia, Italy) (Table 1). Diagnosis was based on current clinical criteria [27]. Informed consent forms were collected for all subjects, and the study was approved by the local ethical committee “Comitato etico Pavia” (protocol number: 0003036/23) and carried out in accordance with the Declaration of Helsinki. Etiological diagnosis of MCI was provided by cerebrospinal fluid (CSF) analysis (Aβ and τ protein levels) or amyloid PET: MCI positive to AD biomarkers (MCI due to AD, 18 subjects—A + T + by CSF analysis) and MCI with negative AD biomarkers (MCI core clinical criteria, 12 subjects). The exclusion criteria were diagnosis of any different neurological or psychiatric condition and secondary causes of cognitive decline such as metabolic, iatrogenic, toxic, and endocrine.

**Table 1:** Demographics, clinical and neuropsychological data

Subjects	
Gender(males/females)	13/17
Age (years)	73 ± 5
MCI +/MCI-	18/12
Neuropsychological scores	
MMSE	27.1 ± 2.3
Verbal long-term memory	0.8 ± 1.1
Visuoconstructional function	2.5 ± 1.4
Visuo-spatial long-term memory	1.7 ± 1.2
Verbal short term memory	3.1 ± 1.0
Semantic fluency	2.5 ± 1.3
Phonological fluency	2.5 ± 1.1
Visual attention and task switching	1.0 ± 1.4
Verbal working memory	3.0 ± 1.1

### Neuropsychological assessment

All subjects underwent a neuropsychological examination assessing the global cognitive function with the Mini-Mental State Examination and a standardized battery of tests to assess different cognitive functions. Neuropsychological tests were grouped into the following cognitive domains, as previously done by our group [21]: verbal short-term memory (digit span forward), verbal working memory (digit span backward), verbal episodic memory (short story test and FCSRT), spatial episodic memory (Rey–Osterrieth complex figure recall), phonological and semantic fluency (Category and Phonemic Fluency tasks) visuo-constructional abilities (Rey-Osterrieth complex figure copy) and visual attention and task switching (trail making test part A and B).

According to the reference norms for the Italian population, raw scores for each test were corrected for age, education and sex. The resulting scores were then converted into equivalent scores (ES), ranging from 0 to 4, with 0 indicating a pathological performance [28]. Table 1 includes demographic, clinical and neuropsychological data.

### MRI and HD-EEG acquisitions

The MRI acquisitions were performed using a 3 T Siemens Skyra scanner with a 32-channel head coil. The protocol was harmonized within the Neuroscience and Neurorehabilitation network, including diffusion weighted imaging (DWI) and resting-state fMRI (rs-fMRI) scans [29].

For DWI data, an axial double-shell single-shot Spin Echo (SE)—Echo-Planar Imaging (EPI) (voxel size = 2.5 × 2.5 × 2.5 mm^3^, TR/TE = 4100/95 ms, two shells with 30 isotropically distributed directions, diffusion weighting of 1000 and 2000s/mm^2^, 7 non-diffusion weighted images (b_0_ images), MB = 2) was performed. Three non-diffusion weighted images were additionally acquired with the reversed phase-encoding direction for distortion correction.

For the rs-fMRI data, an axial Gradient Echo (GE) EPI sequence (voxel size = 3 × 3 × 3 mm^3^, TR/TE = 1270/30 ms, 200 volumes, MB = 2) was set.

For anatomical reference, the protocol included a high-resolution 3D sagittal T1-weighted (3DT1) scan (voxel size = 1 × 1 × 1 mm^3^, TR/TE = 2300/2.96 ms, TI = 900 ms, flip angle = 9°).

HD-EEG data was collected using the Geodesic EEG System (GES) 200 device, which includes a pre-wired headset with 128 HydroCel Geodesic Sensor NetTM (GSN) type electrodes, in accordance with the international 10–20 acquisition system. Electrodes impedances were kept below 10 kΩ using a potassium chloride solution (RPE-ACSISO5). The sampling frequency for signals recording was 1024 Hz. Participants lay in bed and were instructed to remain awake, psychophysically relaxed (no movement), with eyes closed. 15 min resting-state HD-EEG recordings were performed within a 6-months period with MRI acquisitions.

### Preprocessing of DWI, rs-fMRI and HD-EEG data

Preprocessing of diffusion and fMRI data was performed according to Monteverdi et al. [20, 21].

DWI data was denoised and corrected for motion and eddy currents distortions [30] (FMRIB Software Library, FSL). White matter, gray matter (GM), subcortical GM and CSF were segmented from the co-registered 3DT1 image [31] (MRtrix3) and a 30 million streamlines whole-brain anatomically constrained tractography [32] was performed, estimating fibers orientation distribution with multi-shell multi-tissue constrained spherical deconvolution and using probabilistic streamline tractography [33].

fMRI preprocessing was carried out combining SPM12 (Welcome Department of Cognitive Neurology), FSL and MRtrix3 commands in a custom MATLAB script (v2019b, The MathWorks, Natick, Mass). Marchenko-Pastur principal component analysis (MP-PCA) denoising [34] was firstly performed, followed by slice-timing correction, realignment, co-registration to the 3DT1 volume, polynomial detrending, nuisance regression with 24 motion parameters [35] and CSF temporal signal [36], and temporal band-pass filtering (0.008–0.09 Hz).

HD-EEG signals were downsampled at 250 Hz and then filtered within the frequency band from 0.5 to 45 Hz, following international guidelines. Artifacts due to shimming events or heavy movements were manually rejected within EEGLAB toolbox [37]. Following visual inspection, a principal component analysis (PCA) with 64 components was performed to identify and reject the components associated with heartbeat and eye movements [38, 39].

### Structural and functional connectivity

GM parcellation was created including 93 cerebral labels from the Automated Anatomical Labeling (AAL) [40] atlas and 31 cerebellar labels from the Spatially Unbiased Atlas of the Cerebellum and Brainstem (SUIT) [41]. For each subject, structural (SC) and static and dynamic functional connectivity (FC and FCD) matrices were reconstructed applying the parcellation atlas to whole-brain tractography, and BOLD signals extracted from rs-fMRI data, respectively. In particular, the parcellation atlas applied to whole-brain tractography generated two types of connectivity matrices: a distance matrix containing the lengths of the tracts connecting each pair of nodes, and a weight matrix in which the normalized number of streamlines represented the connection strength. Experimental FC and FCD (expFC and expFCD) were reconstructed from rs-fMRI data to capture respectively the synchronous fluctuation of BOLD signals and their spatiotemporal dynamics [42]. The Pearson Correlation Coefficient (PCC) between the time-courses of each pair of nodes was used to compute expFC. After a Fisher's z transformation, the resulting matrix was thresholded at 0.0126 [43]. The expFCD was obtained through two sequential processes: first, FC was calculated using a sliding window of 40 s with incremental shifts of 1TR; second, FCD was estimated as a time-versus-time matrix that included the correlation between the FCs at various time points and the time-evolving dynamics [44]. To identify GM nodes belonging to the six main functional networks of the brain a co-registration between our ad-hoc atlas and Buckner [45] and Yeo [46] functional atlases was performed, identifying: i) integrative networks: default mode network (DMN), frontoparietal network (FPN), limbic network (LN), attention network (AN); ii) motor and sensory networks: visual network (VN), somatomotor network (SMN). The subset of nodes defining each network and their connections were extracted from the whole-brain SC, FC and FCD matrices to generate network-specific matrices.

### Source estimation and power spectral density analysis

For each subject, to combine HD-EEG and MRI data, the length of the HD-EEG signals was reduced to 6 min, matching the length of the rs-fMRI acquisition to ensure consistency across modalities. This is a far more extensive time period than the session lengths generally used in the EEG literature and is sufficient for resting-state spectral estimates and HD-EEG connectivity analyses, whose reliability has been shown to plateau at approximately 6 min of recording [47]. Then, to determine the origin of HD-EEG signals in different brain regions, source estimation was performed using the Brainstorm software. First, the 3DT1 (subject specific anatomical image) was segmented using Freesurfer and imported in Brainstorm. Then, a model of the subject's head was reconstructed using the boundary element method (BEM) for computing the lead field matrix. The cortex mesh for this computation was imported from Freesurfer and down-sampled to a total size of 15,000 vertices. The other three layers of the BEM (scalp, outer skull and inner skull) were computed using 1922 vertices each and a thickness of 4 mm. After that, HD-EEG and MRI were co-registered, first based on the fiducial points (nasion, left and right pre-auricular point) and then refined with the full head shape of the subject. The noise covariance matrix was derived from the post-preprocessed HD-EEG signals before source estimation. The sLORETA (Standardized Low Resolution Electromagnetic Tomography) algorithm was employed to estimate the signal sources and address the inverse problem [26]. The detected sources (i.e., the vertices of the cortical mesh) were arranged according to the brain regions specified by the parcellation atlas previously used for reconstructing the SC/FC matrices. This method made it possible to extract the region-specific HD-EEG time series of the six main functional networks (DMN, LN, AN, FPN, VN, and SMN). Power spectrum (PSD) (0.5–45 Hz) was obtained through Fast Fourier transformation (FFT) of these time-series and spectral power was computed for six frequency bands (delta (0.5–4 Hz), theta (4–8 Hz), alpha (8–12 Hz), beta (12–25 Hz) and gamma (25–45 Hz)). For FFT calculation we used the Welch method, with a window length of 1 s, no overlap and a resolution frequency of 250 Hz. Peak amplitudes (PA) were computed for each frequency band. Spectral band balance or high-frequency/low-frequency ratio was computed as: Hf/Lf = (alpha PA)/(delta PA + theta PA).

### Virtual brain modelling

The Wong-Wang model [10], implemented as Python code, was selected to simulate brain dynamics. This neural mass model simulates neuronal activity in each brain region as a network of excitatory and inhibitory neurons interconnected via NMDA and GABA synapses, offering a representation of the excitatory/inhibitory balance. Within each brain region, the excitatory population presents a recurrent self-excitation (w ^+^) and is connected to the inhibitory one through the excitatory synaptic coupling (J_NMDA_) that is NMDA mediated, while the feedback inhibitory coupling (J_i_) is GABAergic (Suppl. Figure 1). The E/I balance was evaluated computing the E/I ratio = (J_NMDA_ + w ^+^)/J_i_. The SC matrix weights inter-region connections and is scaled by the global coupling (G), which denotes long-range coupling strength between nodes. All model parameters were set as in Deco and colleagues [10], except for the four parameters (i.e., G, J_i_, J_NMDA_, w ^+^) that were optimized to retain information on subject-specific E/I (Suppl. Figure 2). These parameters were combined, and the simulated neuronal activity was fed into the Balloon-Windkessel hemodynamic model to reconstruct BOLD time courses over 6 min and compute simulated FC (simFC) and FCD (simFCD) matrices. ExpFC and expFCD were used as cost functions to optimize model fitting. For FC, Wong-Wang parameters were tuned until the PCC values between the expFC and simFC reached their maximum. In contrast, for FCD, the Kolmogorov–Smirnov distance (KS) was computed and lower KS values indicated higher similarity between expFCD and simFCD. Thus, the optimal simulation led to the highest PCC and the lowest DKS and an overall cost function (Cost) was determined as follows:

$$\mathrm{Cost}\;=\;(1-\mathrm{PCC})\;+\;\mathrm{KS}$$

Lower cost function values reflected the best model fit to both static and dynamic FC data.

### Statistical analysis

Statistical tests were performed using SPSS v21, to analyze i) differences between frequency bands of MCI + and MCI- in different networks; ii) the correlation of HD-EEG and TVB parameters with neuropsychological scores:

i) For each subject, PAs of the frequency bands were averaged across regions belonging to each network, obtaining the mean network-specific PA that were tested for normality (Shapiro–Wilk, *p* < 0.05). Differences between frequency bands PA, Hf/Lf ratios and E/I ratios in the networks of MCI ^+^ and MCI^-^ were assessed with Mann–Whitney tests. Only for the bands presenting a significant difference between the two groups, region-specific PAs were further compared between MCI ^+^ and MCI^-^.
ii) Neuropsychological scores of cognitive domains were compared between MCI ^+^ and MCI^-^ assessing differences through Mann–Whitney tests. Backward regressions were performed to explore whether the variance of neuropsychological scores, either of all patients or of MCI ^+^ and MCI^-^ separately, was significantly explained by HD-EEG or TVB parameters either alone or in combination. Neuropsychological scores in each cognitive domain were considered as dependent variables while the parameters (only HD-EEG, only TVB, HD-EEG and TVB together) derived for each network were used as predictors in a backward approach. The regression algorithm automatically removed one or more predictors to identify which of them significantly (F test, *p* < 0.05) explained neuropsychological scores variance.

## Results

**Fig. 1:**
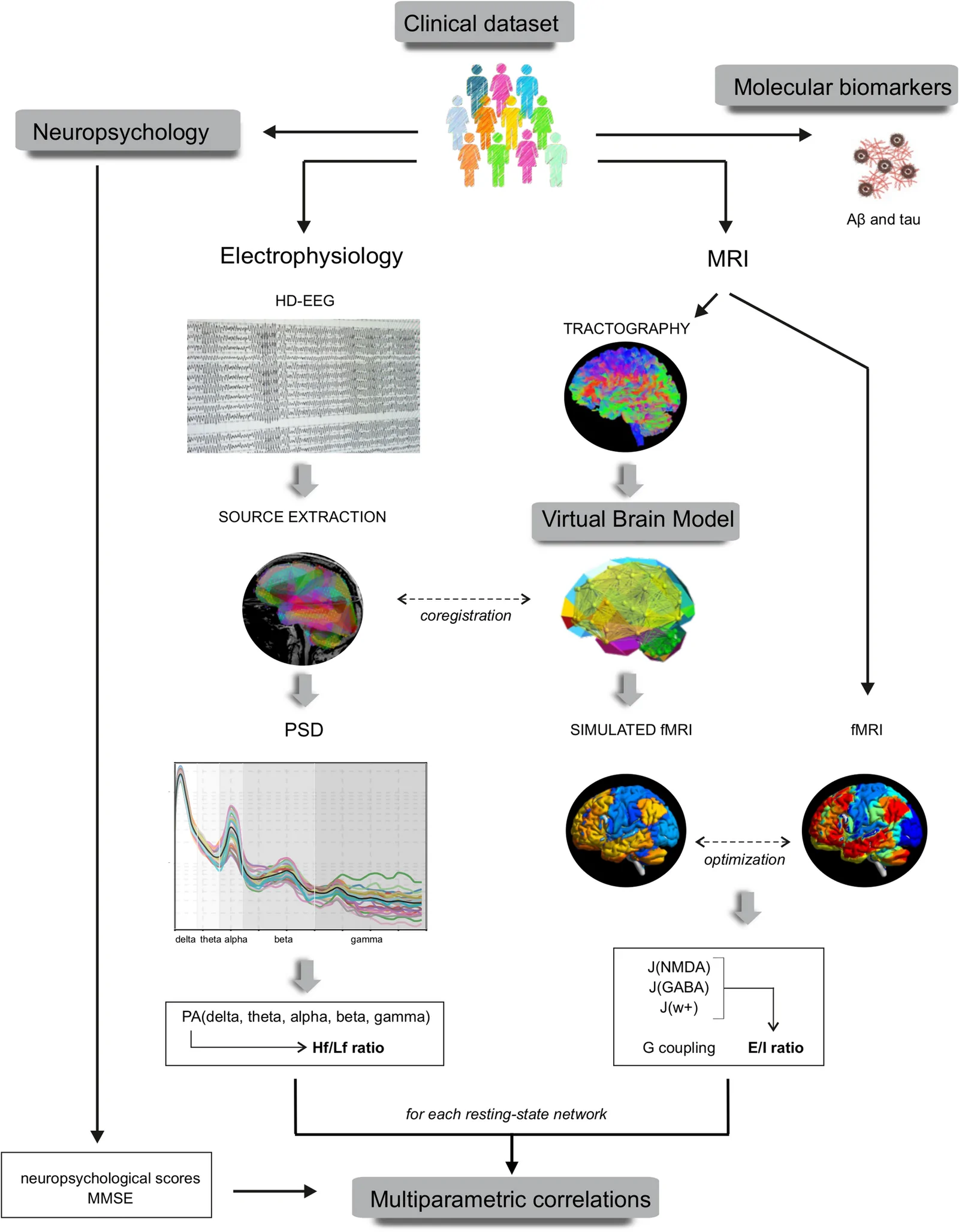
Experimental workflow. Mild cognitive impairment (MCI) patients underwent clinical assessment, the determination of molecular biomarkers (Aβ and tau), neuropsychological testing, and experimental recordings of HD-EEG data, structural MRI data (diffusion weighted images) and functional MRI data (resting-state fMRI). MRI data allowed to model the patient’s brain dynamics. The Virtual Brain (TVB) was reconstructed starting from structural MRI data and simulations were optimized towards functional MRI data extracting a set of parameters describing network communication along with node mechanisms and excitation-inhibition balance. For this analysis, the brain was divided into 6 resting state networks based on fMRI. HD-EEG were used for brain rhythms characterization. EEG source extraction using sLORETA allowed to co-register EEG data on the same brain atlas used for virtual brain modelling. The parameters derived from TVB and HD-EEG were used to capture MCI heterogeneity according to AD biomarker status

**Fig. 2:**
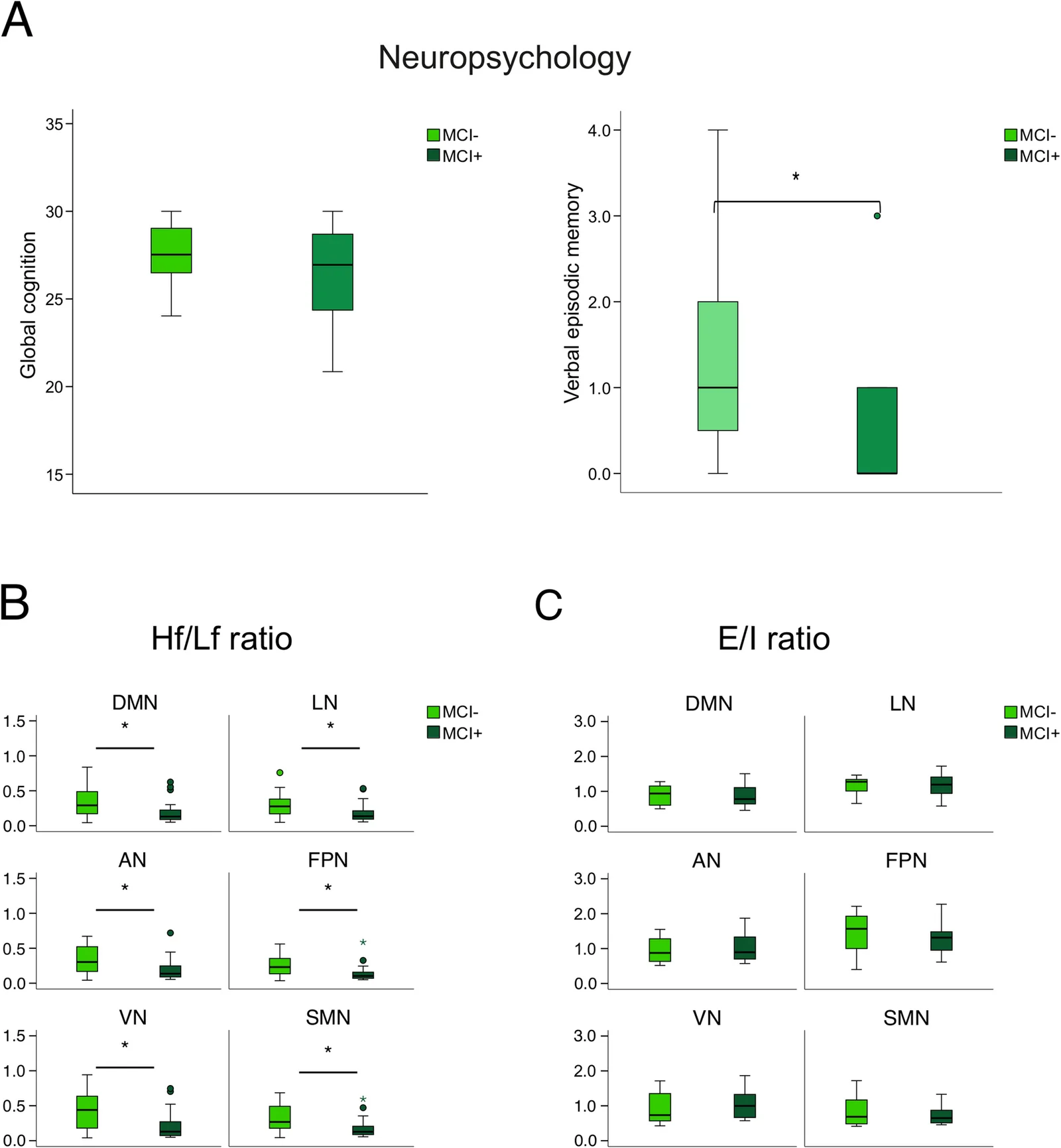
Neuropsychology, Hf/Lf ratio, and E/I balance in MCI. Boxplots show the group level comparison between MCI ^+^ and MCI^-^ parameters. **A** Global cognition and verbal episodic memory scores in neuropsychological tests. Verbal episodic memory is significantly reduced in MCI ^+^, while global cognition does not show any significant difference. **B** High frequency/low frequency (Hf/Lf) ratio derived from HD-EEG in functional resting-state networks. Asterisks show significant differences (*p* < 0.05, Mann–Whitney test), in which the Hf/Lf ratio is always smaller in MCI ^+^ than MCI^-^. **C** Excitation/inhibition (E/I) ratio derived from virtual brain models in functional networks. No significant differences are observed. In this and in the following figures: DMN (default mode network), LN (limbic network), AN (attention network), FPN (frontoparietal network), VN (visual network), SMN (somatomotor network)

The analysis of MCI patients was carried out as shown in Fig. 1. All subjects (30 MCI) were first screened for molecular biomarkers (Aβ and tau) positivity. At the neuropsychological level, MCI^+^ (*n* = 18) did not show significant differences in global cognition (Mann–Whitney *p* = 0.217) compared to MCI^−^ (*n* = 12) but had a lower verbal episodic memory score (Mann–Whitney *p* = 0.04) (Fig. 2A). Virtual brain modelling (TVB) was performed for all the functional resting-state networks identified by fMRI (results are presented here for the DMN, LN, AN, FPN, VN and SMN). HD-EEG analysis was performed with sLORETA to generate source signals in the same regions segmented for MRI and corresponding to TVB nodes. In each of these regions, the PSD peak amplitude (PA) was measured for relevant frequency bands. Finally, these region-matched multimodal and multiparametric data were correlated with patients’ cognitive performance. Two global indicators of circuit function were considered first, i.e., the E/I balance obtained from TVB and the Hf/Lf ratio obtained from HD-EEG. There were no significant differences in E/I, while Hf/Lf was significantly lower in MCI^+^ than MCI^−^ (Fig. 2B,C) (Mann–Whitney, DMN: *p* = 0.045; LN: *p* = 0.045; AN: *p* = 0.042; FPN: *p* = 0.045; VN: *p* = 0.042; SMN: *p* = 0.025).

### Spectral band balance in MCI ^+^ and MCI^-^

**Fig. 3:**
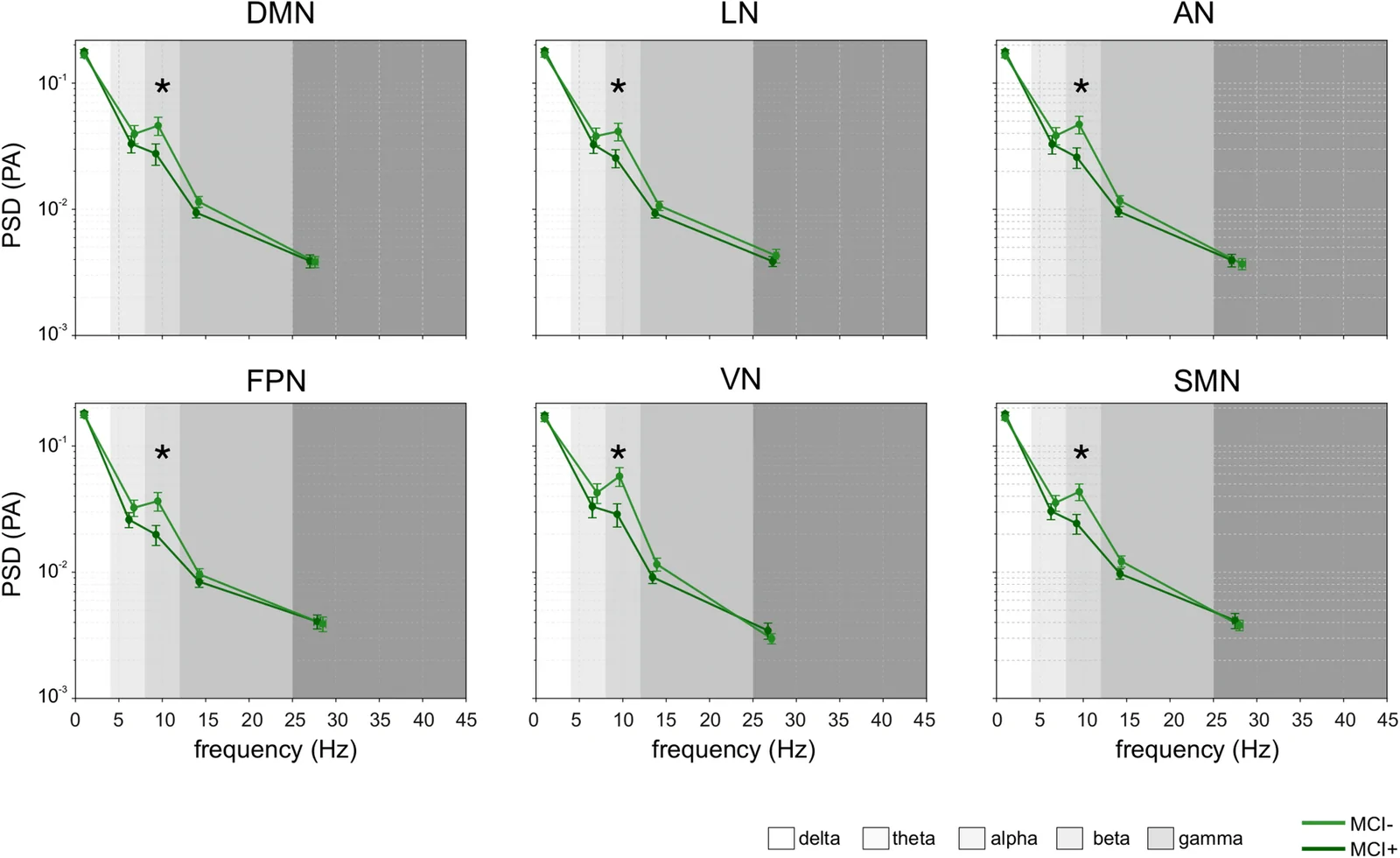
EEG power spectra in MCI. Alpha band peak amplitude (PA) in MCI^-^ and MCI ^+^ in all functional networks. Asterisks show significant differences (*p* < 0.05, Mann–Whitney test), in which the alpha-band PA is always smaller in MCI ^+^ than MCI^-^

**Fig. 4:**
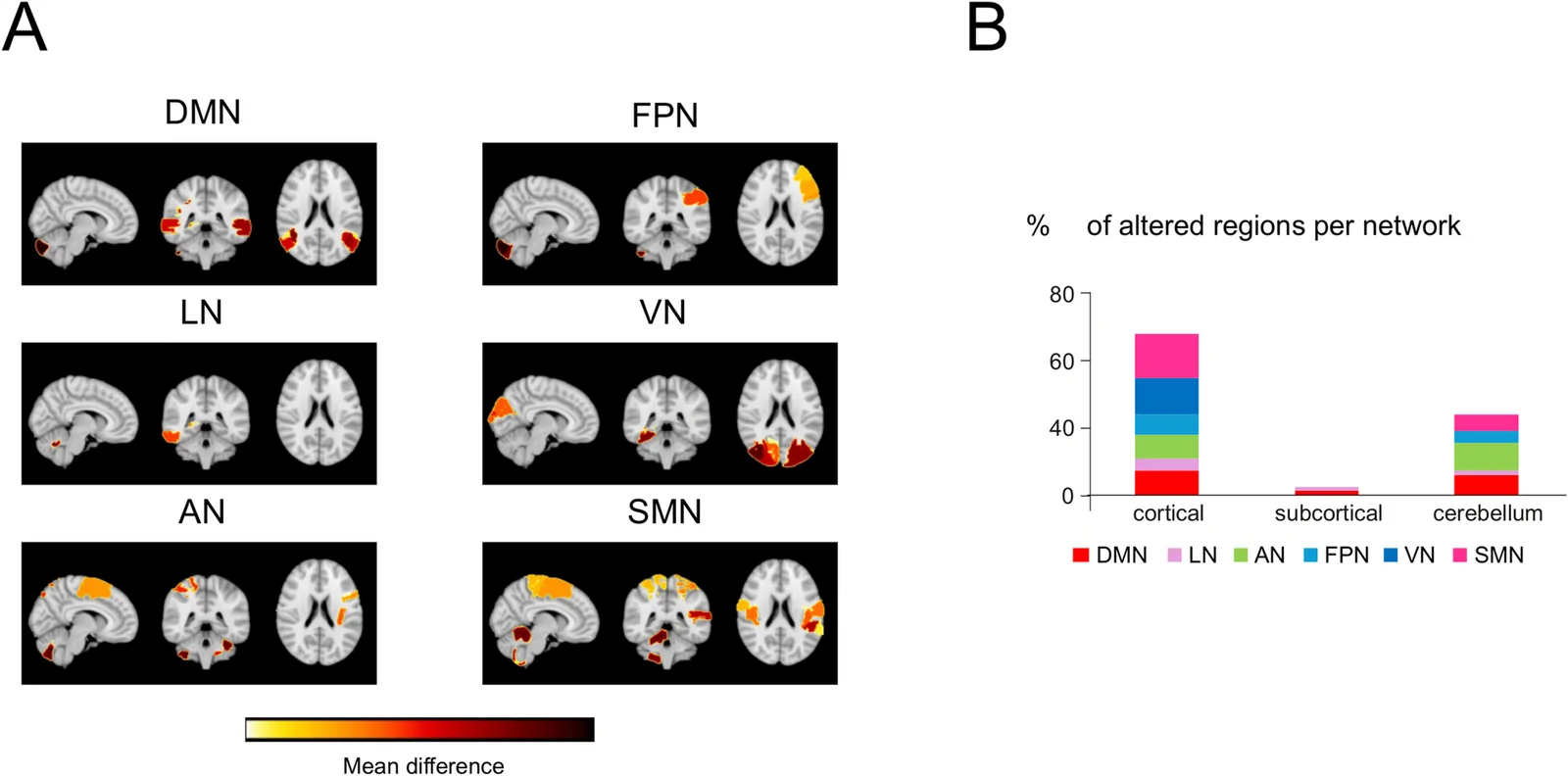
Region-specific alpha band in MCI. **A** Visual representation of cortical, subcortical and cerebellar regions in the functional networks presenting a significant difference in alpha band power between MCI^-^ and MCI ^+^. **B** Barplots show the count (expressed as percentage, 100% is the total of altered regions) of cortical, subcortical and cerebellar regions presenting alpha band significant difference between MCI^-^ and MCI ^+^. It should be noted that most cortical regions are in the DMN, VN, and SMN, while most cerebellar regions are in the AN

In all networks, the power spectra showed distinct peaks (see Fig. 1, Fig. 3) in EEG bands with decreasing PA in the sequence delta, theta, alpha, beta and gamma typical of resting-state recordings [48, 49]. Interestingly, the alpha band PA was significantly lower (Mann–Whitney, DMN: *p* = 0.025; LN: *p* = 0.031; AN: *p* = 0.018; FPN: *p* = 0.034; VN: *p* = 0.018; SMN: *p* = 0.018) in MCI^+^ than MCI^−^ in all networks (Fig. 3). Thus, the lower Hf/Lf balance in MCI^+^ (Fig. 2B) was mostly due to a reduced alpha band. Region-specific analysis (Fig. 4) showed that the alpha band was significantly lower in MCI^+^ in 64% of cortical regions, in 32% of cerebellar regions, and in 3% of subcortical regions (all in the hippocampus) (Fig. 4B). Altered cortical regions were mainly in the SMN, VN and DMN, while cerebellar regions were mainly in the AN.

### Correlation of cognitive scores with TVB and HD-EEG parameters

**Fig. 5:**
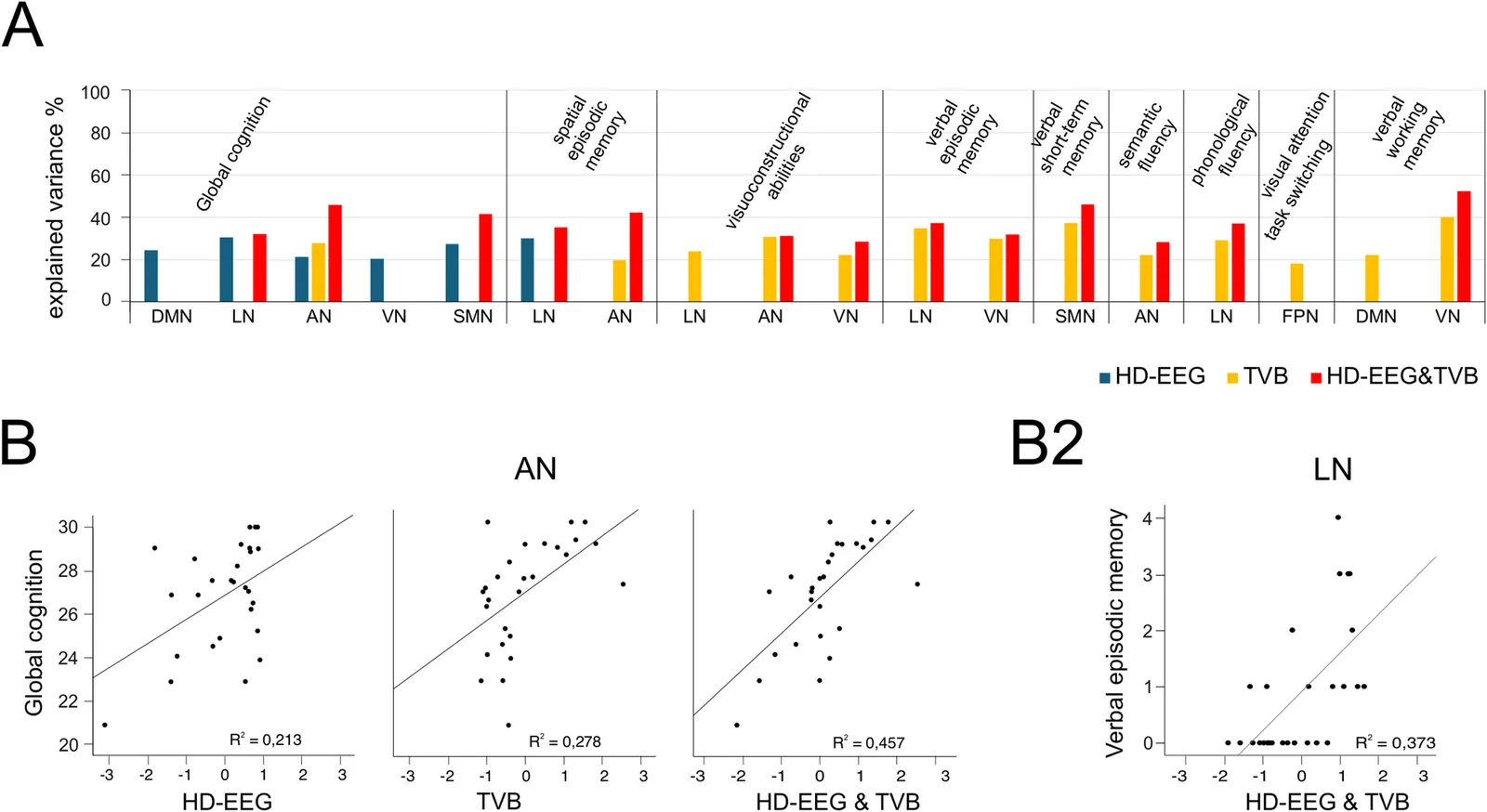
Correlation of cognitive scores with TVB and EEG parameters in MCI. **A** The barplot shows the explained variance (% R^2^) in backward regressions of neuropsychological scores against HD-EEG, TVB, or HD-EEG&TVB parameters in different resting-state networks. All reported values are statistically significant (*p* < 0.05, F-test). HD-EEG parameters alone explain only global cognition, while TVB parameters capture the neuropsychological variance in multiple cognitive domains. The combination of HD-EEG&TVB parameters increases the explained variance in most cognitive domains reaching over 40%. **B1 **The exemplar plots show correlations between global cognition and HD-EEG, TVB, or HD-EEG&TVB parameters in the AN. **B2 **The exemplar plot shows the correlation between verbal episodic memory and HD-EEG&TVB parameters in the LN

The TVB and HD-EEG parameters were correlated using backward regression with neuropsychological scores addressing multiple cognitive domains (Fig. 5A), including global cognition, verbal episodic memory, spatial episodic memory, verbal short-term memory, semantic working memory, visuoconstructional abilities, phonological fluency, verbal fluency, visual attention and task switching.

Considering all MCI patients, TVB parameters significantly explained the neuropsychological variance in multiple cognitive domains, while HD-EEG parameters almost exclusively explained global cognition. The combination of TVB and HD-EEG parameters increased the explained variance in most cognitive domains up to over 40% (Fig. 5B1,B2, Table 2).

**Table 2:** Cognitive domains backward regression results

NPS variable	NETWORK	EEG predictors	explained variance	significance	TVB predictors	explained variance	significance	EEG + TVB predictors	explained variance	significance
MMSE	DMN	theta, alpha	24.4%	0.035						
	LN	theta, alpha	30.5%	0.013				theta, G	32.1%	0.01
	AN	theta	21.3%	0.015	G, Ji	27.8%	0.02	theta, G, Ji	45.7%	0.003
	VN	theta	20.4%	0.018						
	SMN	theta, alpha	27.4%	0.022				theta, alpha, beta, Ji	41.6%	0.015
verbal episode memory	LN				w +, Ji, J_NMDA	34.6%	0.019	theta, w +, Ji, J_NMDA	37.3%	0.03
	VN				G, Ji	29.8%	0.014	beta, G, Ji	31.9%	0.029
visuoconstructional abilities	LN				w +, J_NMDA	23.8%	0.038			
	AN				w +	30.7%	0.003	delta, w +	31.2%	0.011
	VN				J_NMDA	22.2%	0.013	gamma, J_NMDA	28.5%	0.018
spatial episodic memory	LN	beta, alpha, delta	30.00%	0.039				beta, alpha, delta, J_NMDA	35.3%	0.04
	AN				J_NMDA	19.7%	0.02	alpha, delta, G, w +, Ji	42.2%	0.031
verbal short term memory	SMN				w +	37.3%	0.001	alpha, delta, w +	46.1%	0.002
semantic fluency	AN				Ji	22.2%	0.013	alpha, Ji, J_NMDA	28.3%	0.05
phonological fluency	LN				w +, Ji	29.2%	0.016	beta, Ji	37.1%	0.004
visual attention and task switching	FPN				G	18.2%	0.048			
verbal working memory	DMN				G, w +	22.1%	0.05			
	VN				G, w +, Ji	40.1%	0.007	0.007 gamma, G, Ji	52.4%	0.001

The variance explained by the parameters used in backward regressions is calculated with the R2 index and the relative significance of F test (significant

threshold is set at *p* < 0.05). In MCI a different combination of network (default- DMN; limbic-LN; attention-AN; frontoparietal-FPN; visual-VN;

somatomotor-SMN) features significantly explains a percentage of cognitive domains variance

**Fig. 6:**
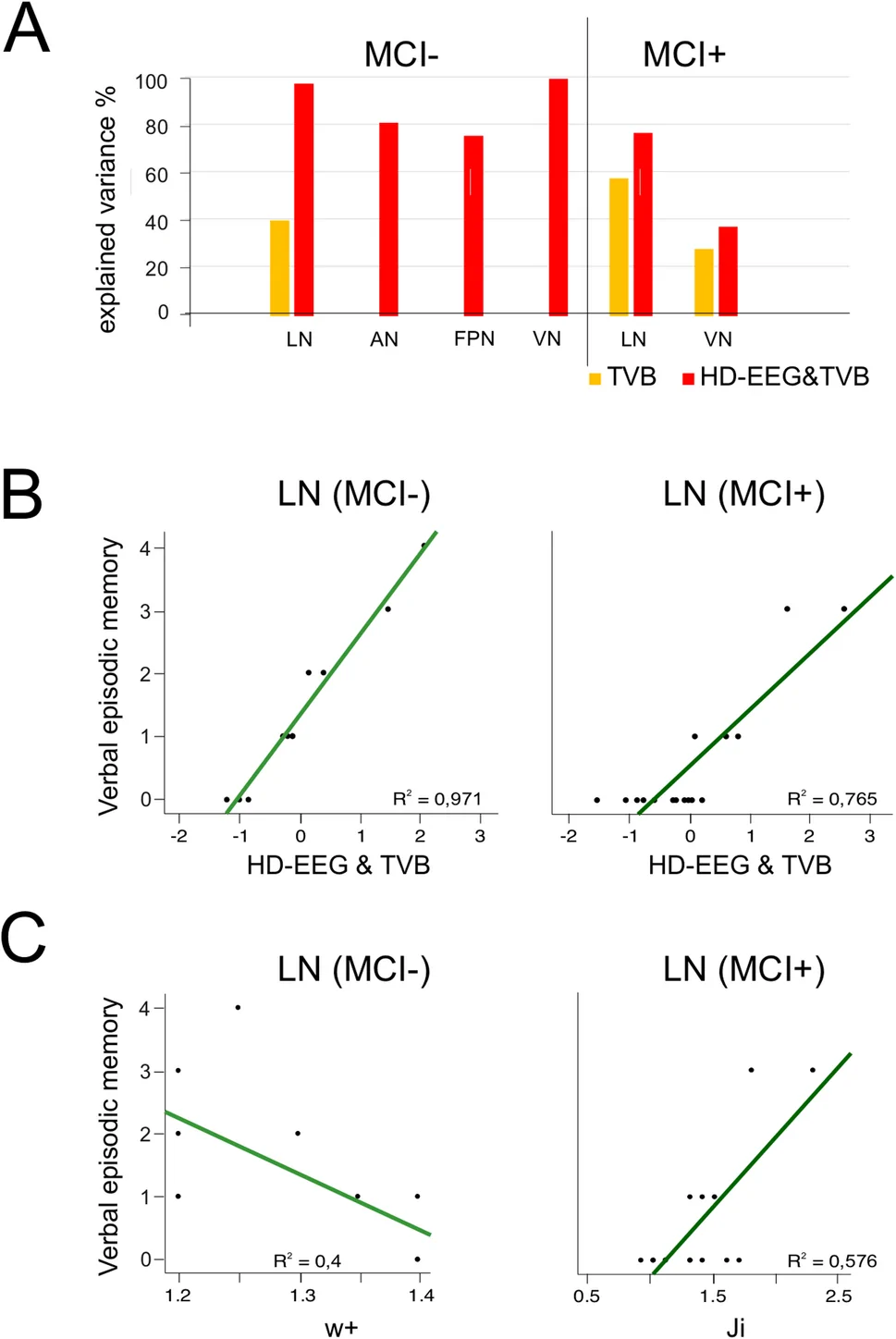
Correlation of cognitive scores with TVB and EEG parameters in MCI ^+^ vs. MCI^-^. **A **The barplot shows the explained variance (% R^2^) of verbal episodic memory in backward regressions against TVB or TVB&HD-EEG parameters in different brain networks. All reported values are statistically significant (*p* < 0.05, F-test). **B** TVB&HD-EEG parameters of the limbic network show a significant correlation explaining more than 90% of verbal episodic memory variance in MCI^-^ and 80% in MCI^ +^.**C** The TVB parameters w ^+^ and J_i_show a significant correlation with MCI^-^ and MCI ^+^ in the LN

Considering MCI^+^ and MCI^−^ separately, TVB parameters alone significantly explained verbal episodic memory variance when extracted from the LN of MCI^−^ and the LN and VN of MCI^+^, while HD-EEG parameters alone did not significantly correlate (Fig. 6A). The combination of TVB and HD-EEG parameters increased the correlations and extended them to AN, FPN, VN of MCI^−^ and to VN of MCI^+^. Parameters extracted from the LN explained more than 90% of verbal episodic memory variance of MCI^−^ and 80% of verbal episodic memory variance in MCI^+^ (Fig. 6B, Table 3). It should be noted that the only TVB parameters of LN that survived in the backward regression were J_i_ for MCI^+^ and w^+^ for MCI^−^ (Fig. 6C).

**Table 3:** Verbal episodic memory backward regression results

biomarkers	NPS variable	NETWORK	TVB predictors	explained variance	significance	EEG + TVB predictors	explained variance	significance
MCI-	verbal episodic memory	LN	w +	40.0%	0.037	delta, theta, alpha,gamma, G, w +, J_NMDA	97.1%	0.025
		AN				delta, theta, alpha, Ji	80.8%	0.024
		FPN				beta, w +, J_NMDA	75.3%	0.016
		VN				delta, theta, alpha, beta, gamma, w +, J_NMDA, Ji, G	99.3%	0.028
MCI +	verbal episodic memory	LN	Ji	57.6%	0.001	delta, beta, alpha, Ji	76.5%	0.002
		VN	Ji	28.2%	0.034	delta, Ji	37.4%	0.048

The variance explained by the parameters used in backward regressions is calculated with the R2 index and the relative significance of F test (significant

threshold is set at *p* < 0.05). In MCI negative and positive to biomarkers (MCI- and MCI + respectively) a different combination of network (limbic-

LN; attention-AN; frontoparietal-FPN; visual-VN) features significantly explains a percentage of verbal episodic memory variance. EEG parameters

alone are not significant predictors of verbal episodic memory performance while some TVB parameters alone are able to significantly explain memory

performance variance of patients, in particular the levels of inhibition (Ji) of the LN for MCI +. EEG and TVB parameters combined as predictors

explain a significant amount of variance (above 90%)

## Discussion

This work reports a multimodal investigation of network parameters in MCI subjects combining virtual brain (TVB) simulations with high-density electroencephalography (HD-EEG) and multi-domain neuropsychological testing. This multimodal approach allowed to generate effective brain digital twins and to bring the analysis from the group to single subject level. MCI subjects were distinguished into MCI^+^ and MCI^−^ depending on the positivity of the molecular biomarkers, Aβ and tau. MCI^+^ had a more severe verbal episodic memory [50, 51] impairment than MCI^−^ and showed a reduced Hf/Lf balance due to a decreased alpha band power. These findings are consistent with literature [52], indicating that our sample was representative of the MCI population. Beyond this, TVB parameters correlated with neuropsychological scores over multiple cognitive domains, indicating their informative capacity about the patient’s state. The combination of TVB with HD-EEG parameters proved superior to individual parameters alone to predict the neuropsychological performance. Further, in the nodes of the limbic network, increased recurrent excitation and reduced inhibitory strength were significantly correlated with the loss in verbal episodic memory, providing new cues for the interpretation of MCI physiopathology.

Hippocampal and cerebellar involvement in MCI network alterations.

The difference in reduction of alpha band power between MCI^−^ and MCI^+^ is consistent with a specific effect of the accumulation of Aβ and tau [3, 4]. The progressive reduction of alpha band power along the AD continuum is well known in literature, pointing out the importance of this electroencephalographic band for monitoring cognitive impairment progression [52]. Interestingly, EEG-based functional connectivity disruptions have been reported also in cognitively healthy individuals with pathological levels of CSF Aβ and tau, providing evidence of EEG changes related to biomarkers positivity even before manifesting significant cognitive decline [23]. Recent studies have shown the link between Aβ and tau and alpha band changes in MCI, either combining EEG acquisitions with PET imaging [3] or revealing a correlation between alpha band power and CSF p-tau and Aβ42 biomarkers levels [4]. Our results support this evidence, suggesting Aβ and tau burden as a key factor contributing to the reduction of alpha activity across all functional networks. Further, thanks to high-density acquisitions followed by source extraction, we were able to identify cortical, subcortical and cerebellar regions presenting alpha changes related to biomarkers positivity in MCI and assign them to the functional networks.

The alpha band reshaping was mainly revealed in cortical regions belonging to DMN, VN and SMN, as well as in hippocampal regions and cerebellar regions belonging to AN. Interestingly, the involvement of DMN, VN and SMN is in line with the spatial patterns of Aβ and tau accumulation across functional networks demonstrated through PET imaging studies [53]: indeed, DMN regions show the highest Aβ accumulation and tau patterns overlap with VN, LN and SMN [53].

The hippocampal involvement in MCI is consistent with the contribution of hippocampal structures to scalp EEG [54] and with reduced hippocampal alpha band activity in MEG studies [55]. Alpha rhythms are widely recognized to reflect the neuromodulatory activity of subcortical ascending systems [4, 56, 57] and the reduction of alpha band activity in the hippocampus may reflect a reduced efficiency of cholinergic neurotransmission and E/I imbalance [57].

The cerebellar involvement in MCI is likely to reflect the impairment of long-range bidirectional communication with the cerebral cortex involving signal processing on the alpha band, e.g., in visuo-attentional tasks [58]. Our study is the first showing alpha band cerebellar changes correlated with Aβ and tau positivity in MCI, in line with the recent observation of Aβ deposition in cerebellar regions [59] and with alteration of cerebello-thalamo-cortical functional connectivity [60].

Multiparametric correlations capture MCI neuropsychological heterogeneity.

Backward regression analysis revealed the relationship between brain network rhythms, E/I profiles and neuropsychological heterogeneity in MCI. Indeed, TVB parameters correlated with MCI performance in multiple neuropsychological domains, while HD-EEG parameters correlated only with global cognitive performance. The combination of HD-EEG and TVB parameters achieved the highest predictive power, explaining up to 40% of the MCI neuropsychological variance in the cognitive domains explored. Interestingly, increased recurrent excitation and reduced inhibitory strength were significantly correlated with the loss of verbal episodic memory. In our previous studies [20, 21] we demonstrated that TVB can explain neuropsychological performance, and we did it in multiple neurodegenerative diseases, encompassing typical and atypical forms of AD, different phenotypes of frontotemporal dementia and now prodromic AD phases, such as MCI. Overall, these studies are supporting the crucial role of E/I parameters in explaining neuropsychological performance.

Digital twin parameters predict network alterations affecting memory performance.

Verbal episodic memory, a hallmark of MCI [50, 51] and in particular of MCI due to AD [61], was the only cognitive domain sensitive to Aβ and tau positivity. Verbal episodic memory disruption reflects disturbances in the ability to learn, store and retrieve information, and has been related to the grey matter volume of medio-temporal regions and hippocampal cortices [62]. TVB parameters of the LN, which includes these critical regions, presented indeed a correlation with memory. In MCI^+^, synaptic inhibition decreased with verbal episodic memory suggesting that the LN inhibitory level is critical to support cognitive performance. This result is of particular interest considering the hypothesis that the evolution of MCI into AD dementia involves neuromodulatory subcortical systems decreasing cortical inhibition and reducing the alpha band power [4]. In MCI^−^, synaptic recurrent excitation increased with the loss of verbal episodic memory, possibly indicating a compensatory mechanism. This is in line with role of recurrent excitation in cortical circuits to support efficient network functioning [63], decision making and working memory tasks [64, 65]. Consistently, we already pointed out in previous studies that higher levels of recurrent excitation occur in the brain of healthy controls compared to patients [20] and in AD simulations strong self-excitation has been suggested to compensate for alterations induced by synaptic loss [66]. The combination of HD-EEG with virtual brain biophysical parameters explained more than 90% of variance for verbal episodic memory scores, confirming the compound origin of memory performance involving both excitatory/inhibitory levels and network electroencephalographic activity acting in concert.

### Study considerations and perspectives

Although a small cohort of patients was enrolled, this study reveals a high correlation between TVB and HD-EEG parameters and neuropsychological scores. The limited impact of the sample size reflects the fact that this study is not based on statistical comparisons but rather on subject-specific analysis ending up in multiparametric correlations. To reduce the risk of overfitting, backward regressions were used to reduce the number of predictors in the statistical models between experimental parameters and neuropsychological scores. In the future, some improvements can be foreseen. First, the extension to a larger cohort is warranted to improve the resolution of individual parameters contributing to digital twin reconstruction. Larger independent cohorts will also be needed to ensure the generalization of the present findings and the reproducibility of the regression models through dedicated validation procedures and cross-validation strategies. Further, in this study Mini-Mental State Examination was used for measuring global cognitive performance in patients instead of Montreal Cognitive Assessment. Both tests are widely used to assess global cognition. Importantly, while the MOCA has higher sensitivity to very mild cognitive decline, the performance of the two tests for screening purposes is comparable and conversion tables for the scores are available [67, 68]. Secondly, the comparison with healthy controls and AD subjects as well as the availability of longitudinal data could help understanding the evolution of the pathology. Thirdly, specific models of cerebellar and cerebrocortical microcircuits should be integrated in virtual brains [69, 70], bringing a detailed representation of physiological microcircuit properties into digital twins. Finally, the capacity of integrating multimodal information (including electrophysiological and topological network constrains [71]) into the TVB generative model and to perform multiparametric optimization of inter- and intra-nodal parameters [72] will largely improve the extraction of information about network alterations in MCI.

## Conclusions

Single-patient digital twins using a combination of TVB and HD-EEG recordings improve our understanding of brain network dynamics in MCI patients. This multimodal and multiparametric analysis enhances the assessment of memory mechanisms and enables to explore non-invasively and in a patient-specific way the relationship between Aβ and tau deposition, E/I balance, electrophysiology, and neuropsychology. Digital twins significantly enhance parameter correlation with neuropsychological scores in multiple cognitive domains, especially with verbal episodic memory. Beyond confirming the correlation of Aβ and tau with a reduced alpha band power, our results reveal major differences in the hippocampal and (for the first time) in the cerebellar nodes according to the positivity or absence of biomarkers. The prospective longitudinal assessment of these subjects will provide new insights about the implications of these parameters in cognitive worsening and in the prediction of conversion to AD dementia, possibly opening additional perspectives for personalized treatment.

## Supplementary Information

Supplementary Material 1.

## Data Availability

All codes used for brain dynamics simulations with TheVirtualBrain are available as a Python code that can be found at https://www.thevirtualbrain.org/tvb/zwei and after free registration on Ebrains platform at https://wiki.ebrains.eu/bin/view/Collabs/tvb-ww-tutorial/. The dataset used and analyzed during the current study is available from the corresponding author Anita Monteverdi, (anita.monteverdi01@universitadipavia.it) upon reasonable request.
