# Supplementary material for: Virtual brain and electroencephalography explain the variance of memory alterations in mild cognitive impairment
Source: Alzheimers Res Ther. 2026 Jun 20;18:210. doi: 10.1186/s13195-026-02114-4 (PMC13613803; doi:10.1186/s13195-026-02114-4)

Supplementary Figure 1| The Wong-Wang Model. Each brain area is represented as a local network composed by excitatory (E) and inhibitory (I) neural populations coupled by pathways of NMDA and GABA synapses. The excitatory synaptic coupling (J_{NMDA}) goes from the excitatory to the inhibitory population and is NMDA mediated, as well as the local recurrent excitation (w_+), while the feedback inhibitory coupling (J_i) is GABAergic. The structural connectivity matrix weights inter-areal connections and is scaled by the global coupling G. Brain dynamics are described by a set of coupled non-linear stochastic differential equations, in which where $r_i^{(E,I)}$ denotes the firing rate of the excitatory and inhibitory populations, $S_i^{(E,I)}$ identifies the average excitatory or inhibitory synaptic gating variables at local area, i, and $I_i^{(E,I)}$ is the input current to the excitatory and inhibitory populations at local area, i.

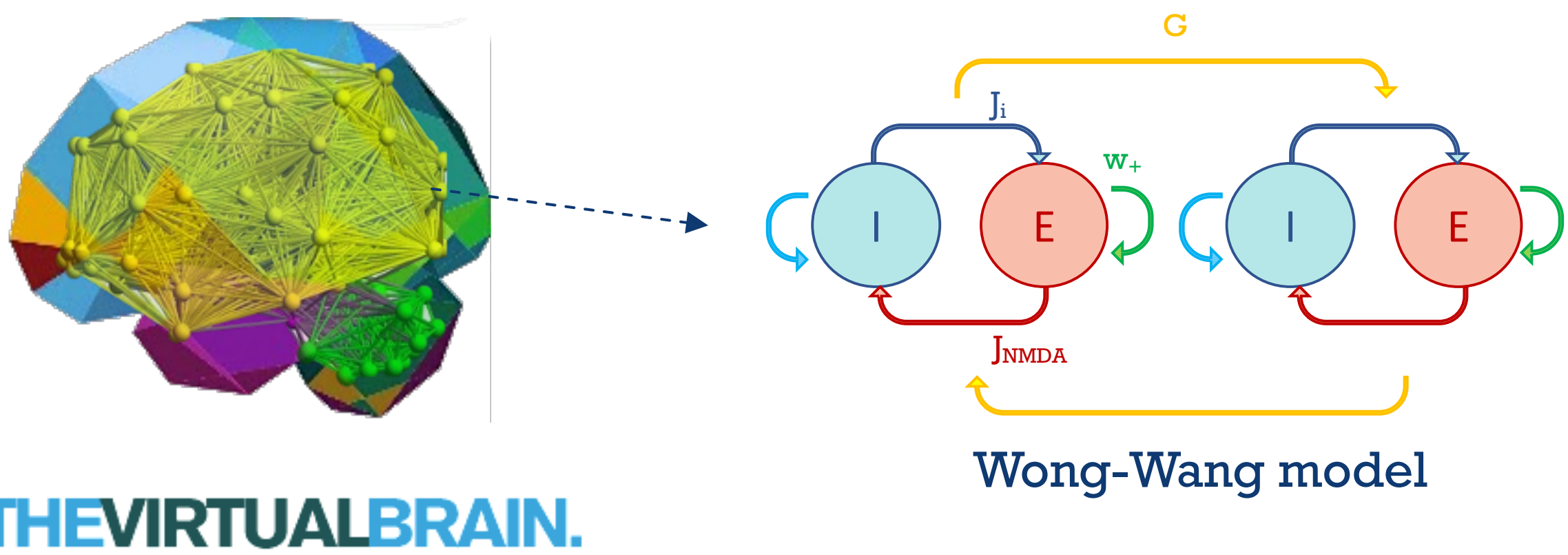

Model Parameters (Wong-Wang)

$$I_i^{(E)} = W_E I_0 + w_+ J_{NMDA} S_i^{(E)} + G J_{NMDA} \sum_j C_{ij} S_j^{(E)} - J_i S_i^{(I)} \tag{1}$$

$$I_i^{(I)} = W_I I_0 + J_{NMDA} S_i^{(E)} - S_i^{(I)} \tag{2}$$

$$r_i^{(E)} = H^{(E)}\left(I_i^{(E)}\right) = \frac{a_E\, I_i^{(E)} - b_E}{1 - \exp\left(-d_E\left(a_E I_i^{(E)} - b_E\right)\right)} \tag{3}$$

$$r_i^{(I)} = H^{(I)}\left(I_i^{(I)}\right) = \frac{a_I\, I_i^{(I)} - b_I}{1 - \exp\left(-d_I\left(a_I I_i^{(I)} - b_I\right)\right)} \tag{4}$$

$$\frac{d\, S_i^{(E)}\,(t)}{dt} = -\frac{S_i^{(E)}}{\tau_E} + \left(1 - S_i^{(E)}\right)\gamma r_i^{(E)} + \sigma\, \upsilon_i\,(t) \tag{5}$$

$$\frac{d\, S_i^{(I)}\,(t)}{dt} = -\frac{S_i^{(I)}}{\tau_I} + r_i^{(I)} + \sigma\, \upsilon_i\,(t) \tag{6}$$

Supplementary Figure 2| Parameter space exploration. Parameter space exploration is conducted in two steps: first, G and J_i are optimized with the other two parameters (J_{NMDA} and w_+) at their standard value; then J_{NMDA} and w_+ are optimized with G and J_i fixed at their optimized value. Heat maps represent the value of the cost function obtained with different parameters combinations. Optimal parameters are the ones leading to the lowest cost function between simulated and experimental data.

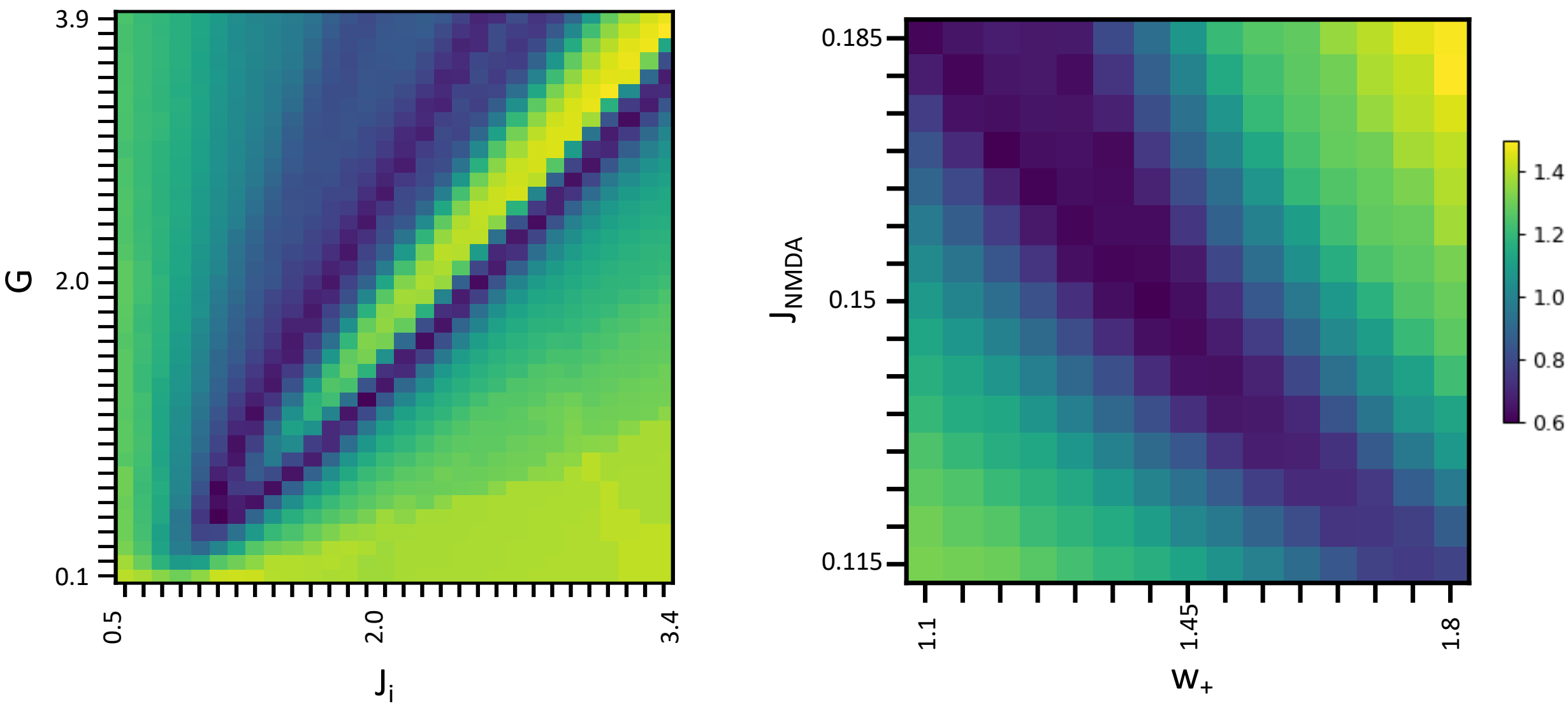

Supplement: Supplementary file 1 [file 13195_2026_2114_MOESM1_ESM.pdf]
